# Hippocampal Local Field Potentials Encode Continuous Flight Speed in Homing Pigeons via Complementary Gamma and Theta Signatures

**DOI:** 10.3390/ani16162569

**Published:** 2026-08-18

**Authors:** Long Yang, Xin Guo, Aimin Tao, Zhihui Li

**Affiliations:** 1 School of Electrical and Information Engineering, Zhengzhou University, Zhengzhou 450001, China; longyang_zzu@163.com (L.Y.); guoxin02@gs.zzu.edu.cn (X.G.); tao676743059@gs.zzu.edu.cn (A.T.); 2 State-Owned Luoyang Dancheng Radio Factory, Luoyang 471000, China; 3 Henan Key Laboratory of Brain Science and Brain-Computer Interface Technology, Zhengzhou 450001, China

**Keywords:** hippocampal formation, representation, navigation, theta, decoding

## Abstract

Although the role of the mammalian hippocampus in representing locomotor speed has been widely investigated, how the avian hippocampus represents continuous flight speed under free-flight conditions in the outdoor environment remains unclear. In this study, we used homing pigeons as a model system and synchronously recorded hippocampal formation (HF) local field potentials (LFPs), global positioning system (GPS) trajectories, and inertial measurement unit (IMU) data during natural homing flights. We aimed to determine whether and how the avian HF encodes flight speed. Flight-speed-related neural features were extracted from both frequency-domain and time-domain signals, including the 50–70 Hz power spectral density (PSD) ratio and theta-demodulated amplitude (DAmp). We then constructed models for discrete flight-speed state decoding and continuous flight-speed prediction. The results showed that the 50–70 Hz PSD ratio in the HF was significantly negatively correlated with flight speed, whereas DAmp was significantly positively correlated with flight speed. Both features exhibited consistent speed-related trends across different spatial release sites. Support vector machine (SVM)-based classification showed that PSD, DAmp, and their combined features could effectively decode four flight-speed states, including non-flight, low-speed, medium-speed, and high-speed states, with the combined features achieving the best performance. Further Gaussian process regression (GPR) analysis demonstrated that the combined features predicted continuous flight speed more accurately than either single feature. These findings provide evidence that the avian hippocampal formation encodes continuous flight speed during natural navigation through the complementary integration of frequency-domain and time-domain features, extending the known role of the avian hippocampal formation from static spatial mapping to dynamic self-motion representation.

## Simple Summary

Survival in complex natural environments depends on an animal’s ability to continuously monitor its own movement. While the role of the mammalian hippocampus in encoding locomotor speed is well established, how the avian hippocampus represents speed during natural flight remains largely unknown. In this study, we recorded neural signals from the hippocampal formation of homing pigeons during real-world homing flights. We found that hippocampal local field potentials contain two complementary features that represent flight speed: the 50–70 Hz power spectral density ratio and the theta-demodulated mean amplitude. These features not only enabled accurate classification of distinct flight speed states, but also supported continuous speed prediction through combined features. Our findings demonstrate that the avian hippocampal formation provides a robust neural representation of flight dynamics, underscoring its role in processing complex movement variables during natural navigation. Beyond its scientific implications, this work may also inform the sport of pigeon racing, where understanding the neural basis of homing speed could have practical applications.

## 1. Introduction

The hippocampus (Hp) is a central structure for spatial memory and navigation. Classical theories propose that it constructs a “cognitive map” of the environment [1,2,3], encoding spatial variables such as position, boundaries, and head direction [4,5]. Navigation, however, is inherently dynamic. To continuously update spatial information and guide navigational behaviour, animals must integrate self-motion signals, including movement speed and direction, through a mechanism known as path integration [6,7,8]. Accumulating evidence further indicates that the mammalian Hp also plays an important role in integrating and representing dynamic variables, such as locomotor speed [9,10,11].

In the rodent Hp, running speed is strongly and positively correlated with both the amplitude and frequency of theta-band activity [12]. In parallel, locomotor speed systematically modulates gamma oscillations in hippocampal CA1: the absolute power of low-gamma oscillations (25–55 Hz) is negatively correlated with running speed, whereas that of high-gamma oscillations (65–100 Hz) is positively correlated with running speed [13]. Electrical stimulation of the hippocampal CA1 region can evoke contractions of the contralateral hindlimb muscles, and hippocampal lesions can also alter locomotor speed [14]. A major advance in understanding hippocampal speed coding came with the identification of “speed cells” in the rodent Hp [9]. These neurons increase their firing rate linearly with running speed, independent of spatial location or head direction, and provide a continuous signal that scales with instantaneous velocity. This speed signal is thought to be critical for path integration. Their discovery has reshaped the classical view of the hippocampus as a purely spatial map, positioning it instead as a multi-dimensional navigational system that tracks both position and self-motion. The intrinsic relationship between theta oscillations and speed modulation has further advanced our understanding of hippocampal speed-coding mechanisms and provided new perspectives on hippocampal function during dynamic navigation [15].

Nevertheless, most studies of neural speed representation have been conducted under highly controlled conditions, such as linear tracks or open-field arenas. Although these paradigms enable precise measurement, they involve relatively simple movement patterns and therefore provide limited insight into natural behaviour [16]. Consequently, how the hippocampus encodes continuously changing movement speed under naturalistic conditions remains poorly understood. This gap is even more pronounced in non-mammalian species, particularly birds.

Homing pigeons provide an ideal model for investigating the neural mechanisms of animal spatial navigation because of their highly stable and reproducible homing ability [17,18]. They are capable of long-distance navigation and high-speed flight in three dimensions, with homing pigeons returning from distances of several hundred kilometers and, in some cases, from as far as approximately 1600 km away [19]. Recent advances in wireless neural recording and multimodal behavioural tracking have enabled simultaneous recording of hippocampal local field potentials (LFPs) and high-resolution behavioural data from freely flying pigeons [16]. This provides a unique opportunity to examine how the hippocampus encodes continuous and highly dynamic self-motion speed under naturalistic conditions. Although the avian brain differs from the mammalian brain in many anatomical respects, accumulating evidence suggests that the avian hippocampal formation (HF) and the mammalian Hp share comparable functional principles for spatial navigation, including the representation of place and head direction [20,21,22]. Whether this cross-species functional similarity extends to the representation of speed during large-scale, highly dynamic flight, however, remains an important open question.

Despite these motivations, three fundamental questions remain unresolved. First, does the avian hippocampus encode flight speed? Second, how do hippocampal LFPs represent continuously varying flight speed during natural flight (self-directed, unconstrained flight in the open air under real-world conditions)? Third, can speed-related hippocampal signals support the decoding and prediction of continuous flight speed? To address these questions, we combined wireless hippocampal LFP recordings with synchronized high-resolution behavioural tracking in homing pigeons during natural homing flights from unfamiliar release sites. We tested two central hypotheses: first, that hippocampal LFP activity contains stable and decodable information about flight speed; and second, that speed-related information is represented through the complementary contribution and integration of multiple frequency-domain and time-domain features, rather than through a single linear coding channel. By testing these hypotheses under naturalistic, large-scale flight conditions, we aim to extend the understanding of hippocampal speed coding beyond conventional laboratory paradigms and to explore its potential cross-species conservation in birds.

## 2. Materials and Methods

### 2.1. Subjects

Six adult homing pigeons of either sex were used in this study. The birds were approximately 15 months old and weighed 400–450 g. All pigeons had been reared from an early age in a loft located in the attic of the School of Electrical and Information Engineering. Throughout the study, the position of the home loft was kept constant to maintain a stable homing target for the birds. The pigeons were trained once daily for approximately 30 min, which involved free flight around the loft area. Water, a commercial pigeon grain mix (consisting of corn, wheat, peas, and sorghum), and mineral-based grit (containing calcium, grit stone, and trace minerals) were provided at 17:00 each day.

### 2.2. Experimental Procedures

Homing flight experiments were conducted at 08:00 and 17:00 each day, corresponding to periods of relatively high pigeon activity and relatively stable solar elevation. To minimize environmental interference, experiments were performed only under clear weather conditions, with no precipitation and wind speeds below level 3 on the Beaufort scale. Each pigeon completed no more than two homing trials per day, with an interval of at least 6 h between trials, to reduce potential effects of fatigue or stress on behaviour and neural signal recording.

**Figure 1:**
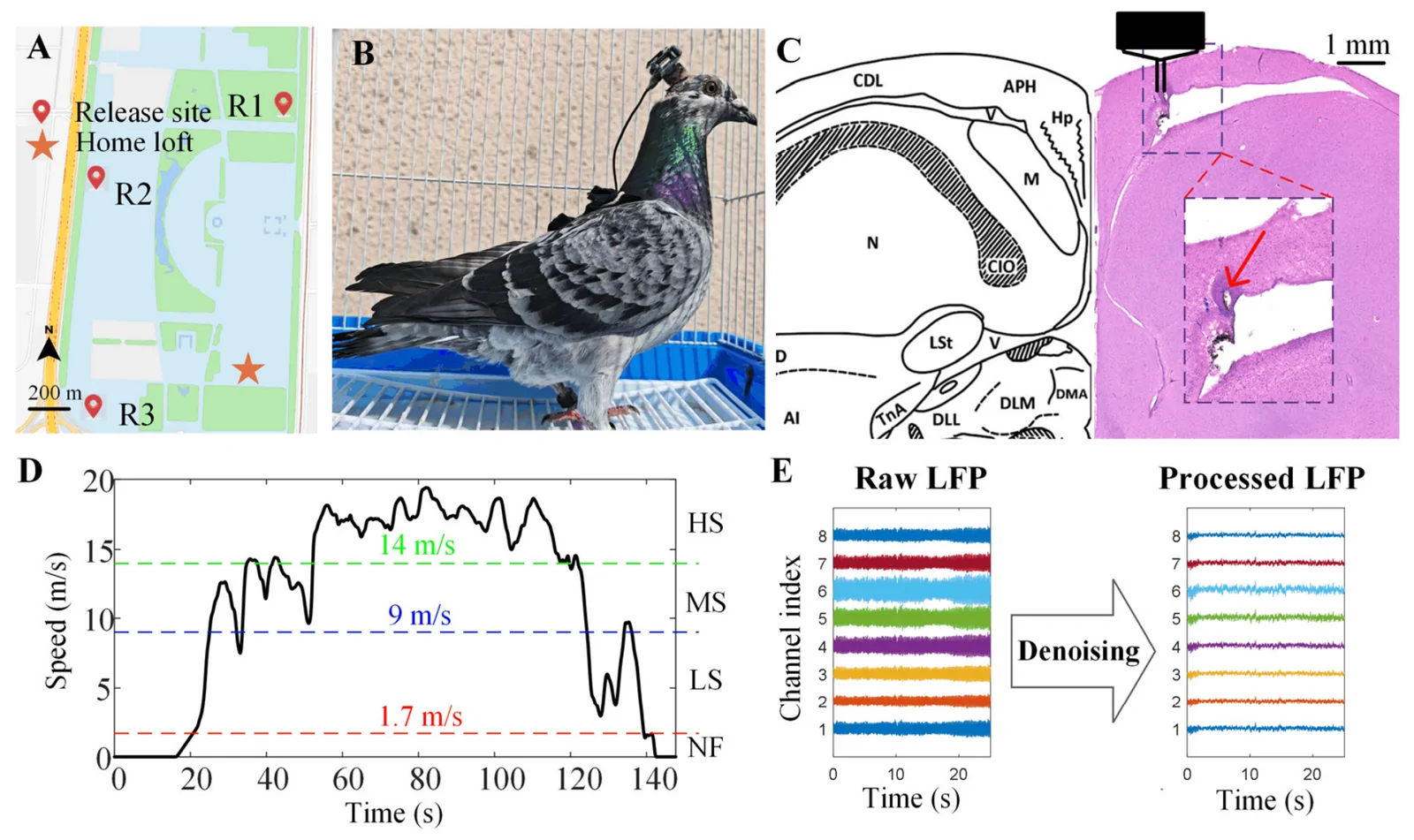
Experimental design and signal preprocessing. (**A**) Schematic illustration of the experimental environment, showing the three release sites located in different directions relative to the home loft. (**B**) Pigeon carrying the experimental recording device. (**C**) Schematic illustration of the HF electrode implantation site. The red arrow indicates the position of the electrode tip. (**D**) Schematic illustration of the four flight-speed states (NF, non-flight state; LS, low speed state; MS, medium speed state; HS, high speed state). (**E**) Representative raw LFP waveforms and denoised LFP waveforms after preprocessing.

To examine the influence of release direction on homing navigation while maintaining environmental diversity and experimental control, three release sites were selected in open areas approximately 800–1500 m from the loft (Figure 1A). These sites were located in open areas to the north, northwest, and southwest of the home loft, respectively, and were selected to be free of nearby tall buildings, dense tree cover, or other prominent visual landmarks that could serve as local navigational cues. The terrain surrounding each site was relatively flat, with low vegetation, and the areas were not regularly accessed by pedestrians or vehicles, thereby minimising environmental disturbance during experiments. Before the experiment began, one of the three release sites was randomly selected. The pigeons were then placed in a custom-made dark transport box and transported to the release site along an indirect route. During transport, the container was subjected to multiple detours, direction changes, and rotations to prevent the pigeons from using inertial or path-integration cues to infer the outward journey. Upon arrival at the release site, the pigeon was fitted with a previously developed wearable data-recording device [23,24] (Figure 1B), allowed to habituate for 1 min, and then released. Neural signals and behavioural data were recorded synchronously during the homing flight. A total of 90 homing flights were analyzed in this study, with 15 flights per pigeon.

### 2.3. Surgery

Prior to surgery, all pigeons showed no marked changes in body weight. Pigeons were anaesthetised (2% sodium pentobarbital, 0.2 mL/100 g) and secured in a stereotaxic apparatus. A 2 × 4 tungsten microelectrode array was implanted into the HF. Using the stereotaxic atlas of the pigeon brain, the electrode implantation site targeting the HF was determined using the following coordinates (relative to bregma): AP 4.50–6.50 mm; ML 0.5–1.5 mm; DV 1.00–1.50 mm [25]. Electrode locations were confirmed by histological verification, as shown in Figure 1C. Full surgical and post-operative details are provided in Supplementary Material. All animal care and experimental procedures were approved by the Life Science Ethics Committee of Zhengzhou University and were conducted in accordance with the Guide for the Care and Use of Laboratory Animals of Zhengzhou University.

### 2.4. Data Acquisition

Neural and behavioural data were recorded using a wearable data-recording device (Figure S1) [24]. The device weighed 13.6 g and consisted of a neural signal acquisition module, a global positioning system (GPS) module and an inertial measurement unit (IMU). Detailed specifications of the recording device and installation procedures are provided in Supplementary Material. Data acquisition began 1 min before release and continued until the pigeon returned to the home loft. During each trial, LFP signals, GPS trajectories and IMU-derived movement data were recorded synchronously.

### 2.5. Typical Flight-Speed State Classification

The relationship between avian flight speed and the mechanical power required for flight follows an approximately U-shaped curve, defining biologically meaningful reference speeds such as the minimum-power speed (approximately 8–9 m/s in pigeons) and the maximum-range speed (approximately 14–16 m/s) [26,27,28]. Based on these considerations, flight speed during homing was divided into four flight-speed states, as shown in Figure 1D: non-flight state (NF), defined as v < 1.7 m/s; low-speed flight state (LS), defined as 1.7 m/s ≤ v < 9 m/s; medium-speed flight state (MS), defined as 9 m/s ≤ v < 14 m/s; and high-speed flight state (HS), defined as v ≥ 14 m/s.

### 2.6. Signal Preprocessing

Flight speed was derived from the GPS data: horizontal speed was computed as the distance between successive GPS fixes divided by the corresponding time interval and smoothed with a 1 s moving-average filter; segments with poor satellite coverage or abrupt position discontinuities were excluded.

Discrete wavelet transform was used for baseline drift removal, effectively separating drift in low-frequency approximation components while preserving neural information in high-frequency details [29]. Large-amplitude motion artefacts in the neural signals were removed using an algorithm based on multivariate empirical mode decomposition (MEMD), which is an adaptive, data-driven method that decomposes multichannel signals into intrinsic mode functions (IMFs). Three-axis acceleration signals recorded by the IMU served as reference channels: IMFs whose amplitude envelope was strongly correlated with the IMU reference were identified as motion-related and removed from all LFP channels. This approach takes advantage of the fact that motion-related noise tends to affect multiple recording channels simultaneously. Representative LFP traces before and after preprocessing are shown in Figure 1E. The effectiveness of this preprocessing pipeline was validated by comparing time-frequency energy maps before and after artifact removal, which confirmed successful suppression of movement-related noise while preserving continuous neural oscillations [30].

### 2.7. Power Spectral Analysis

LFPs were analyzed using MATLAB 2020a (The MathWorks Inc., Natick, MA, USA). The LFP signals were first band-pass filtered between 1 and 200 Hz. PSD was then estimated for LFP segments corresponding to the typical flight-speed range of pigeons, defined here as 1–20 m/s, using an autoregressive (AR) model and the Burg algorithm [31,32,33,34]. For analyses of speed-related PSD changes and flight-speed state decoding, PSD estimation was performed using a 1 s sliding window with a step size of 0.5 s, and spectral power was calculated within the frequency range of 1–120 Hz. To quantify the relative contribution of each frequency band, the PSD ratio was calculated by dividing the absolute power within a given frequency band by the total power of the corresponding time window. For continuous flight-speed prediction, the same PSD-ratio calculation was applied to neural signals segmented using 0.5 s sliding windows with 50% overlap, as described in the decoding and prediction section.

### 2.8. Demodulation Analysis

A complex principal component analysis-based demodulation approach was used to examine the relationship between hippocampal LFPs and flight speed. Theta-band rhythms were treated as carrier rhythms that may contain information related to neural activity. Accordingly, demodulation was used to extract theta-band features potentially associated with flight speed [4,35,36,37,38,39]. The LFP signals were first band-pass filtered between 4 and 12 Hz to isolate theta-band activity. The filtered signals were then processed using a complex Morlet wavelet with zero-phase filtering to obtain the instantaneous amplitude and phase, as described in Equation (1).


(1) $$\psi \left(t\right)=\frac{1}{\sqrt{\pi f_{b}}}e^{-2\pi if_{c}t}e^{\frac{-t^{2}}{f_{b}}}$$

In this equation, the center frequency $f_{c}$ was set to 7 Hz, and the bandwidth $f_{b}$ was set to 0.002.

**Figure 2:**
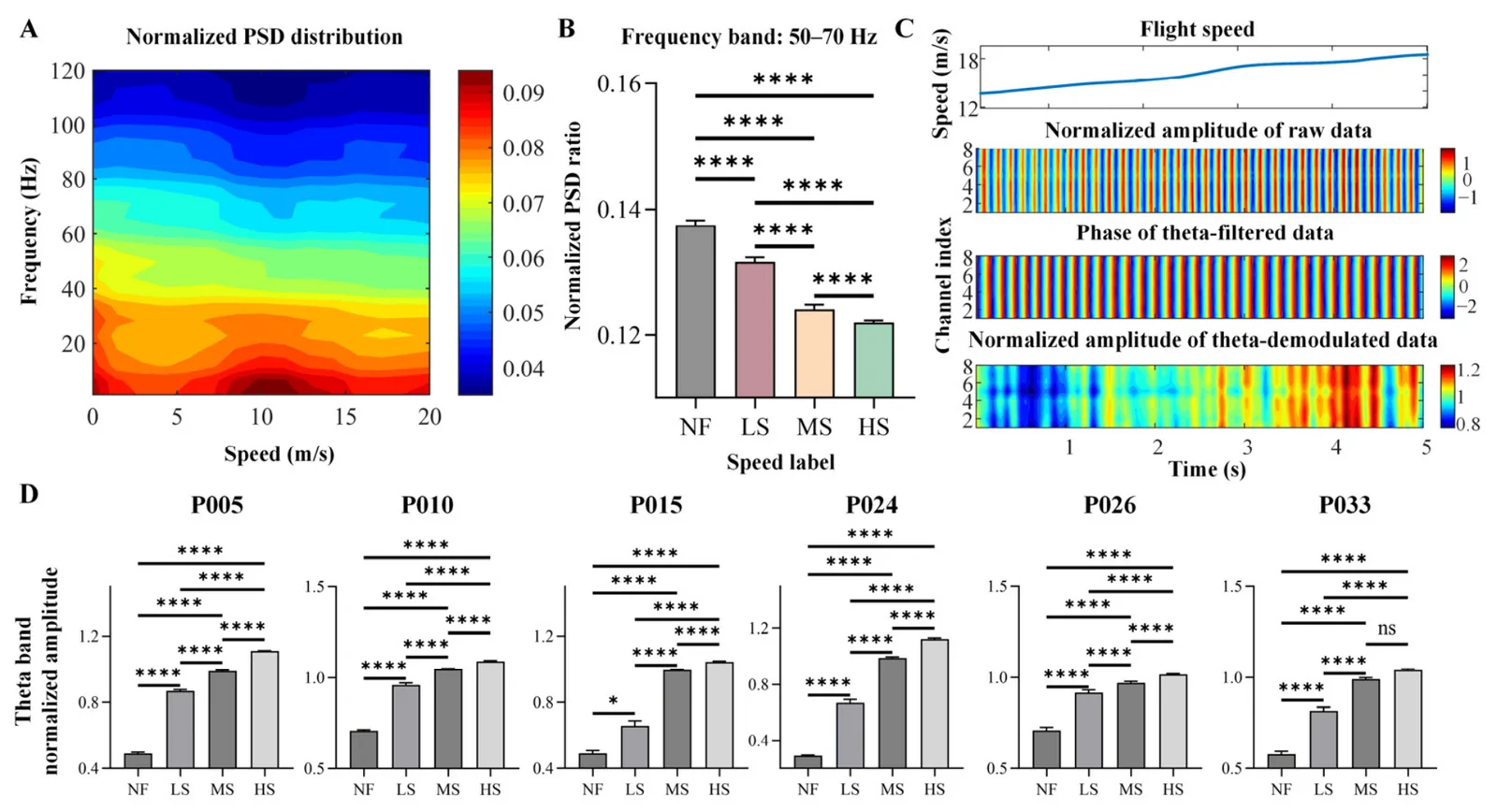
Flight-speed-related hippocampal LFP features in homing pigeons. (**A**) Example of the normalized PSD across frequency bands as a function of flight speed. (**B**) The 50–70 Hz LFP PSD ratio decreased as flight speed increased. (**C**) Example of theta-band demodulation of LFP signals during a flight with varying speeds. (**D**) Changes in normalized theta-demodulated amplitude across different flight-speed states in individual pigeons. * indicates *p* < 0.05, **** indicates *p* < 0.0001, ns indicates *p* > 0.05.

Complex principal component analysis was then applied to the filtered multichannel LFP signals to extract the first principal component, which represented the dominant oscillatory component shared across recording channels. Based on this component, the multichannel theta-band LFP signals were demodulated using Equation (2) to remove the phase of the theta carrier oscillation and obtain the relative phase and amplitude after theta demodulation, as shown in Figure 2C.

(2) $$X_{d}\left(t\right)=X_{t}\left(t\right)e^{-\phi_{c}\left(t\right)i}$$

In Equation (2), $X_{t}(t)$ denotes the complex theta-band LFP signal obtained after Morlet wavelet transformation, $\phi_{c}(t)$ denotes the instantaneous phase of the first complex principal component extracted from the multichannel theta-band signals, and $X_{d}(t)$ denotes the demodulated signal.

For each flight trial, the normalized demodulated amplitude was first calculated according to the procedure described above. For analyses of speed-related feature changes and flight-speed state decoding, flight speed and mean theta-demodulated amplitude (DAmp) were calculated using 1 s sliding windows with a step size of 0.5 s.

### 2.9. Decoding and Prediction Methods

Discrete flight-speed state decoding and continuous flight-speed prediction models were constructed separately. For flight-speed state decoding, the four flight-speed states—non-flight state (NF), low-speed flight state (LS), medium-speed flight state (MS) and high-speed flight state (HS)—were used as classification labels. The 50–70 Hz PSD ratio, DAmp and their combined features were used as model inputs. A support vector machine (SVM) classifier with a Gaussian kernel was used for classification [40,41]. Classification performance was evaluated using 10-fold cross-validation [42,43,44], with classification accuracy used as the primary performance metric.

Continuous flight-speed prediction was conducted using a 0.5 s window length and 50% overlap to extract average speed and neural features. The Gaussian process regression (GPR) was selected and used an exponential kernel to describe correlations among samples in the feature space, and the kernel parameters were estimated by maximizing the marginal likelihood [45]. Prediction performance was evaluated using the cumulative distribution of absolute prediction errors (CDF) and the root mean square error (RMSE). CDF describes the cumulative probability of prediction errors; a steeper slope indicates smaller errors and higher precision. RMSE quantifies the average deviation between predicted and true values; smaller RMSE indicates better prediction accuracy.

### 2.10. Statistical Analysis

Experiments were conducted only under clear weather conditions with wind speeds below Beaufort level 3; weather was thus controlled as an inclusion criterion rather than modelled as a covariate. Multiple trials per pigeon were treated as repeated measures in our statistical analyses. Both sexes were included; given the limited sample size, sex was not modelled as a statistical factor.

All statistical analyses were performed using GraphPad Prism 9.0 (The GraphPad Software, Boston, MA, USA). Data are presented as the mean ± standard deviation (SD). Data normality was verified using the Shapiro–Wilk test, and homogeneity of variances was assessed using Bartlett’s test. For comparisons among more than two groups, one-way analysis of variance (one-way ANOVA) was used when the data met the assumptions for parametric testing. For non-parametric multiple-group comparisons, Dunn’s multiple comparison test was used. For paired comparisons between two related groups, paired *t*-tests were performed. Statistical significance was set at *p* < 0.05. Significance levels were denoted as follows: ns, *p* > 0.05; *, *p* < 0.05; **, *p* < 0.01; ***, *p* < 0.001; and ****, *p* < 0.0001.

## 3. Results

### 3.1. Representation of Flight Speed by Hippocampal LFPs

To determine whether the avian HF is involved in the representation of flight speed, we analyzed the PSD of hippocampal LFPs during flights at different speeds. The PSD distribution across frequency bands at different flight speeds is shown in Figure 2A. The PSD ratio in the 50–70 Hz band gradually decreased as flight speed increased. In contrast, the theta-band PSD ratio was relatively higher when flight speed was approximately 10 m/s, whereas differences in PSD ratios at other speeds were less pronounced. To quantify these changes, we calculated the 50–70 Hz PSD ratio in the HF across different flight-speed states (Figure 2B). This analysis revealed a significant monotonic decrease in the 50–70 Hz PSD ratio with increasing speed, with significant differences between all speed states (**** *p* < 0.0001, Dunn’s test). To further examine the relationship between theta-band activity and speed representation in pigeons, we used complex principal component analysis-based demodulation to analyze the relationship between HF LFPs and flight speed from the time-domain perspective. The theta-band demodulation procedure is shown in Figure 2C. Prominent theta rhythmic activity was observed in the raw LFP signals during flight, and the extracted phase showed consistent phase information across recording channels. Therefore, theta-band oscillations were treated as carrier oscillations, and demodulation was used to extract theta-band information potentially associated with flight speed. The normalized demodulated amplitude increased with flight speed. Statistical analysis of the mean theta-demodulated amplitude in the HF across flight-speed states showed that the NF had significantly lower theta-demodulated amplitude than all flight states across all individual pigeons (Figure 2D). Meanwhile, it was observed in all individuals that as the speed increased, the normalized demodulated amplitude also exhibited a significant increasing trend. Significant differences were observed among the LS, MS and HS (*p* < 0.05, Dunn’s test).

### 3.2. Stability of Flight Speed Representation Across Release Sites

**Figure 3:**
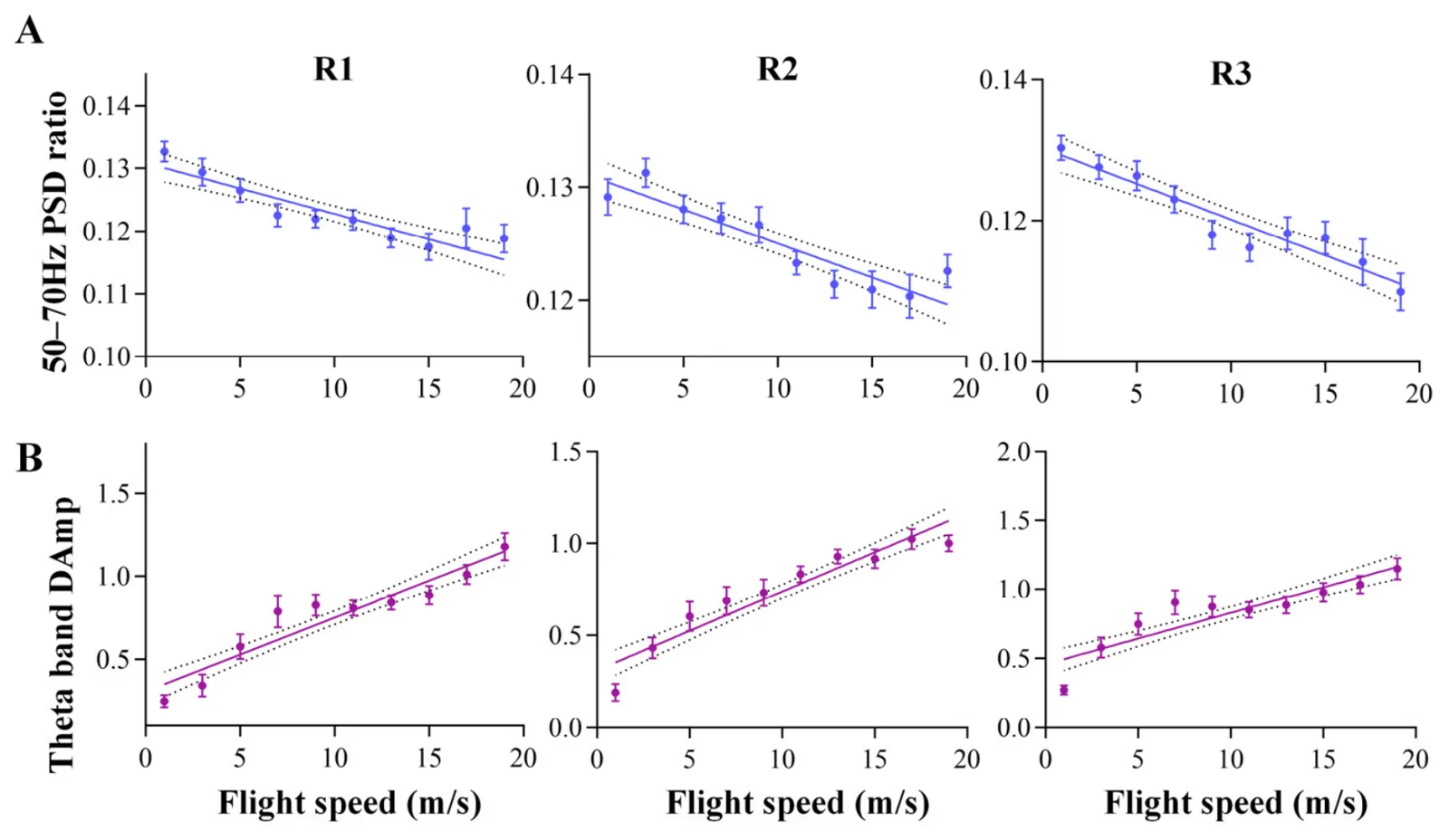
Stability of flight-speed-related hippocampal neural features across release sites. (**A**) Relationship between the 50–70 Hz PSD ratio and flight speed across different release sites. (**B**) Relationship between theta-demodulated amplitude and flight speed across different release sites.

To determine whether flight-speed-related hippocampal neural features were affected by release-site direction or differences in homing trajectory, we analyzed the relationships between flight speed and two neural features, the PSD ratio and DAmp, across the three release sites. Figure S2 shows homing trajectories of pigeons released from three spatially distinct sites. The 50–70 Hz PSD ratio decreased with increasing flight speed at all three release sites (Figure 3A), indicating that the relationship between this frequency-domain feature and flight speed was consistent across different release-site conditions. Analysis of theta-demodulated amplitude further showed that DAmp increased with flight speed under the R1, R2 and R3 conditions (Figure 3B). Together, these results indicate that both the 50–70 Hz PSD ratio and theta-demodulated amplitude exhibited consistent speed-related trends across release sites, suggesting that flight-speed-related hippocampal LFP features were stable across different spatial release conditions.

### 3.3. Decoding Flight Speed State from Hippocampal LFP Features

**Figure 4:**
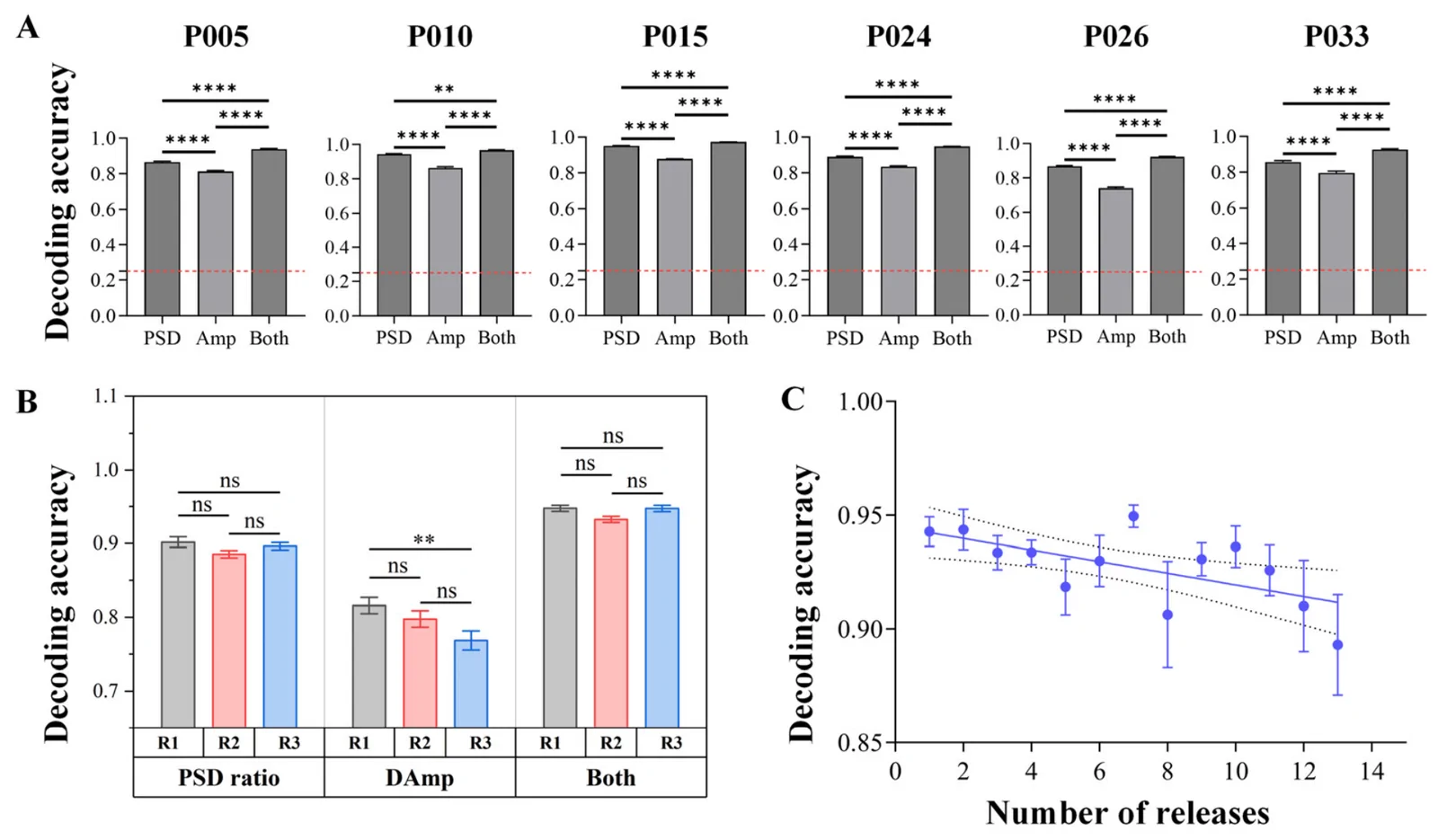
Flight-speed state decoding based on hippocampal LFP features. (**A**) Flight-speed state decoding accuracies based on the PSD ratio, theta-demodulated amplitude and combined features across individual pigeons. The red dashed line indicates the chance level. (**B**) Comparison of decoding accuracies based on the three feature types across different release sites. (**C**) Changes in flight-speed state decoding accuracy based on combined features as the number of release trials increased. ** indicates *p* < 0.01, **** indicates *p* < 0.0001, ns indicates *p* > 0.05.

To further determine whether speed-related neural features in the HF could effectively decode flight-speed states in pigeons, we used three types of input features for SVM-based classification: the 50–70 Hz PSD ratio, DAmp, and their combined features. The results showed that decoding accuracies based on all three feature types were clearly above chance level in all pigeons, indicating that HF neural activity contained information that could distinguish among different flight-speed states (Figure 4A). Further comparison among feature types showed that the combined features achieved higher decoding accuracy than either single feature in each individual pigeon. In addition, the decoding performance based on the PSD ratio was significantly higher than that based on DAmp across all individuals (** *p* < 0.01 and **** *p* < 0.0001, one-way ANOVA). These results indicate that combining the PSD ratio with theta-demodulated amplitude effectively integrated distinct types of neural information in the pigeon HF, thereby improving flight-speed state decoding.

We next compared decoding performance across different release sites (Figure 4B). The results showed that neither the PSD ratio nor the combined features exhibited significant differences in decoding accuracy across different release sites. The accuracy of DAmp feature decoding showed only slight differences between R1 and R3, with no significant differences observed between the other release sites (ns *p* > 0.05; ** *p* < 0.01, Dunn’s test). These results indicated that these features were relatively stable across different release-site conditions. In addition, the combined features outperformed either single feature across all release sites. Further analysis showed that the decoding accuracy of the combined features exhibited a slight decreasing trend as the number of release trials increased. This result suggests that neural signal stability may have been affected to some extent by the duration of electrode implantation. Nevertheless, overall decoding performance remained high, with an approximate decrease of only 5%, and the final mean decoding accuracy still reached 89.3% (Figure 4C). This value was substantially higher than the chance level for four-class classification, indicating that the combined features maintained stable and robust decoding performance throughout the experimental period.

### 3.4. Multi-Domain Feature Fusion Enhances Continuous Speed Prediction

**Figure 5:**
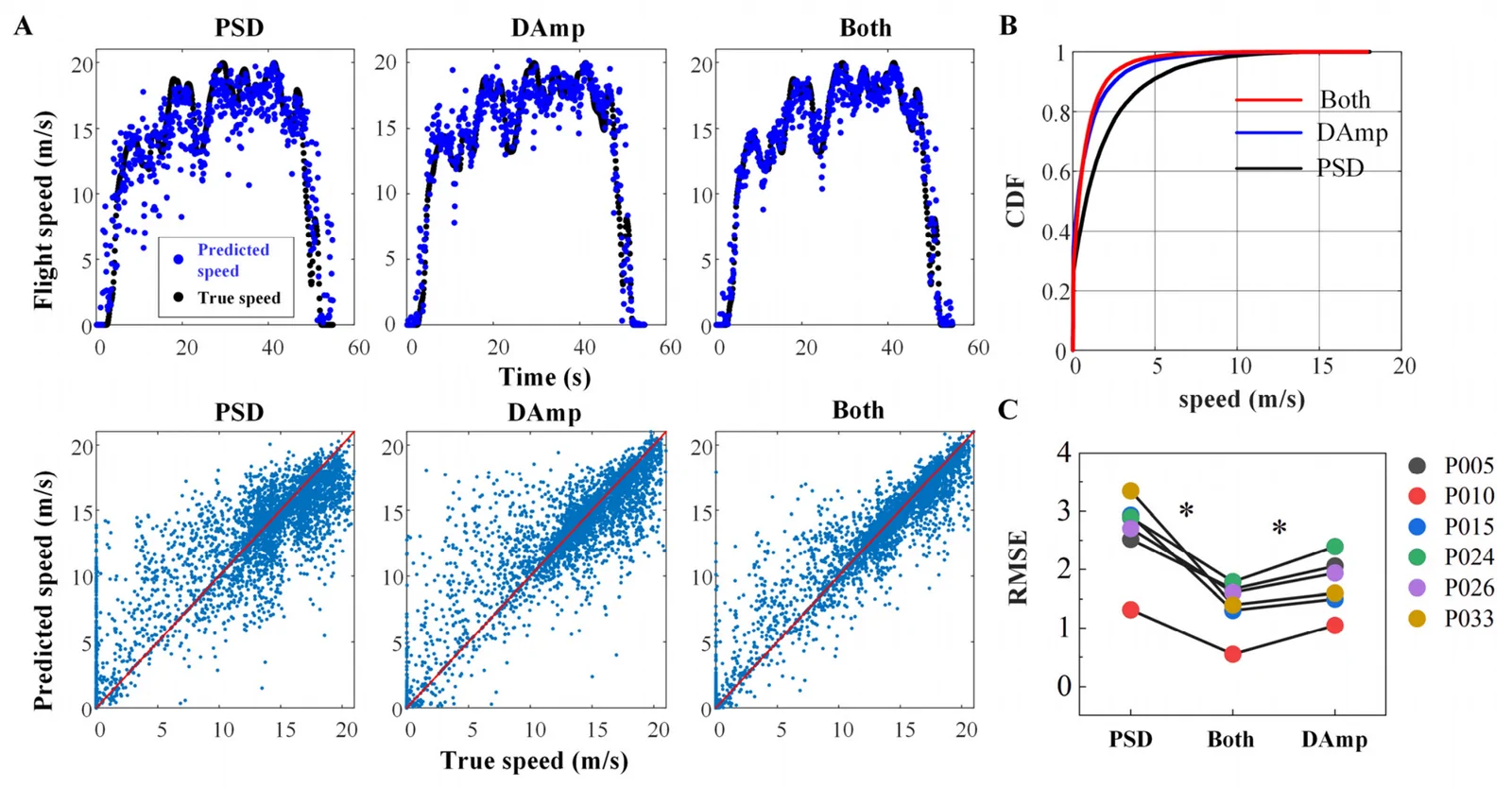
Continuous flight-speed prediction based on hippocampal LFP features. (**A**) Example of continuous flight-speed prediction. The upper panels show actual and predicted flight speeds over time during one homing flight of pigeon P026. The lower panels show the correspondence between actual and predicted flight speeds across all experimental samples. (**B**) Cumulative distributions of prediction errors based on different neural features across all pigeons. (**C**) Comparison of RMSE values for continuous flight-speed prediction based on different neural features across all pigeons. * indicates *p* < 0.05.

To evaluate the ability of hippocampal LFP features to predict dynamic changes in continuous flight speed, we used GPR to predict the continuous flight speed of pigeons during homing flights. Figure 5A shows an example prediction from one homing flight of pigeon P026. The results showed that all three feature types could track the temporal variation in actual flight speed to some extent, with the combined features producing predicted speeds that were closer to the actual speeds. In the scatter plots comparing actual and predicted flight speeds, predictions based on the combined features were more concentrated around the diagonal line, indicating better prediction performance. In comparison, predictions based on DAmp showed slightly weaker agreement with the diagonal line. Predictions based on the PSD ratio were more widely dispersed around the diagonal line, particularly near 0 m/s, indicating poorer prediction performance. These results suggest that the combined features provided a better prediction of continuous flight speed.

Comparison of the cumulative distributions of absolute prediction errors across all experimental samples showed that the error distribution curve for the combined features was shifted towards the lower-error range (Figure 5B), indicating that the combined features yielded smaller prediction errors than either the PSD ratio or DAmp alone. In other words, the fusion of both neural features produced more accurate speed predictions across the entire range of flight speeds. Analysis of the RMSE values across pigeons further showed that the RMSE values obtained using the combined features were significantly lower than those obtained using either the PSD ratio or DAmp alone (Figure 5C, *p* < 0.05, paired *t*-test). Together, these results indicate that both the PSD ratio and DAmp contain information related to continuous flight-speed dynamics, and that integrating the two feature types further improves the accuracy of continuous flight-speed prediction.

## 4. Discussion

In this study, we investigated how hippocampal LFPs represent homing flight speed in pigeons under natural flight conditions. By combining discrete classification of continuous flight speed into four flight-speed states with continuous regression-based prediction, we identified two major findings. First, the PSD ratio in the hippocampal 50–70 Hz sub-band was significantly negatively correlated with flight speed. Second, the mean theta-demodulated amplitude in the time domain was significantly positively correlated with flight speed. Based on these neural features, we further decoded flight-speed states and predicted continuous flight speed. Together, these findings reveal a multidimensional coding mechanism for continuous movement variables in the HF during natural navigation.

Previous studies have shown that, in the rodent hippocampus, power across the theta range (6–12 Hz) is positively correlated with running speed [12,15]. This is consistent with our finding that the time-domain mean theta-demodulated amplitude in the avian HF increased with flight speed. A recent review has further emphasized that the functional connectivity patterns of the avian hippocampus are broadly consistent with those of the mammalian hippocampus, supporting its role in spatial navigation [20]. The avian hippocampus also appears to act as an integrative hub that combines sensory information with internal state signals to support diverse behaviours [20]. These findings suggest that hippocampal representation of speed may be an evolutionarily conserved function across vertebrates. At the same time, previous studies have reported that increased running speed in rats induces a marked upward shift in the center frequency of hippocampal gamma oscillations. Specifically, the absolute power of low-gamma activity (30–52 Hz) is negatively correlated with running speed, whereas the absolute power of higher-frequency activity (65–240 Hz) is positively correlated with running speed [13]. In light of these findings, we speculate that, when pigeons accelerate during homing flight, gamma oscillations involved in hippocampal information integration may shift towards higher frequencies. Such frequency modulation may redistribute spectral energy across frequency bands, leading to a relative decrease in the power ratio of the original mid-to-high-frequency 50–70 Hz band. From the perspective of spectral power, this result further supports the sensitivity of hippocampal oscillations to movement speed.

An important finding of this study is that hippocampal speed representation was highly consistent across different spatial release directions. This suggests that speed encoding was not primarily driven by external environmental features [10], but instead may reflect an intrinsic and fundamental representation of self-motion information within the HF. Such environment-independent representation may allow pigeons to obtain continuous and robust path-integration feedback in complex outdoor environments, thereby helping maintain the accuracy of their cognitive map. Classical cognitive map theory emphasizes hippocampal representations of relatively static spatial variables, such as position and boundaries, which are often environment-dependent [46]. In contrast, the cross-site consistency of speed-related neural features observed in the present study suggests that the hippocampus also represents dynamic self-motion variables in a relatively environment-independent manner. These results may reflect a functional division of information processing in the hippocampus during navigation: context-dependent spatial variables may be used for localization and environmental identification, whereas context-independent self-motion variables may support continuous displacement estimation.

Although flight speed is a continuous physical variable, it often exhibits “staged” characteristics from behavioural and bioenergetic perspectives. Avian flight power follows the classic U-shaped curve [26,27,28], providing a biological rationale for discretizing flight speed into distinct states. In this study, we employed a progressive analytical framework ranging from discrete speed-state decoding to continuous speed regression, aiming to characterize hippocampal speed encoding at different granularities. Flight-speed state analysis served as an initial “existence test”, demonstrating that hippocampal neural features could significantly distinguish among different speed states. The results showed that the PSD ratio provided better representation of discrete flight-speed states than the theta-demodulated feature, leading to stronger state-decoding performance. This suggests that energy redistribution in higher-frequency gamma activity may reflect global dynamical switching of hippocampal networks across different metabolic ranges and behavioural states. Consistent with this interpretation, gamma oscillations in mammalian hippocampal studies are often regarded not as direct representations of specific behavioural events, but rather as state indicators that support transitions of neural networks into distinct computational modes [47]. One possible, though speculative, interpretation is that changes in gamma-band energy partly reflect macroscopic state transitions of hippocampal circuits across behavioural modes.

However, the ability to distinguish among discrete flight-speed states alone is not sufficient to demonstrate that the hippocampus encodes the continuous magnitude of speed. In the continuous regression analysis, we found that the mean theta-demodulated amplitude was better suited to tracking dynamic changes in flight speed and showed stronger performance in continuous flight-speed prediction. This finding is highly consistent with theoretical expectations regarding the functional role of theta rhythms in the mammalian hippocampus. Previous work has suggested that hippocampal theta oscillations may optimize the trade-off between sampling rate and coding precision, thereby enabling efficient information transmission and coding [48]. Within this theoretical framework, gamma oscillations fall within a higher-frequency regime. In the high-noise and highly dynamic context of natural flight, gamma-band activity may show reduced signal-to-noise ratio and less reliable phase locking, making it more suitable as a state indicator than as a precise tracker of continuous variables. In contrast, theta oscillations, owing to their favourable position in the speed–precision trade-off, may be naturally suited for encoding movement parameters that change continuously and require high temporal resolution. Thus, our results are consistent with, though they do not directly test, the prediction of the theoretical framework proposed by Amil et al. [48] that theta oscillations may serve as a preferred carrier for encoding continuous self-motion variables.

We further found that speed decoding and prediction based on combined features were significantly better than those based on either single feature. This result suggests that hippocampal representation of flight speed does not rely on a single linear coding channel, but instead emerges from the integration of multiple frequency bands and feature dimensions. This transition from the limitation of a single feature dimension to the benefit of multidimensional feature integration is consistent with the complex computational logic of the hippocampus [49,50].

Several limitations of this study should be addressed in future work. First, correlation does not imply causation. The observed correlations between hippocampal neural features and flight speed do not prove that hippocampal activity constitutes an internal representation of flight speed; these signals may also reflect activity related to motor output or sensory feedback. Future studies could combine chemogenetic or optogenetic approaches to selectively inhibit or activate hippocampal circuits, thereby establishing causal links [51,52]. Second, during natural flapping flight, flight speed may covary with wingbeat frequency. Therefore, some of the observed neural feature changes may reflect proprioceptive feedback associated with flapping movements rather than flight speed per se [53,54,55]. Future studies could further dissociate these factors using powered gliding paradigms or virtual-reality-based experimental designs. Third, the present study was based only on LFP recordings and did not incorporate single-neuron spike activity. Future studies combining single-cell-resolution recordings may characterize speed tuning at a finer neural scale and determine whether specialized ‘speed cells’ exist for encoding flight speed in the avian HF. Fourth, the sample size of this study was limited (n = 6), although each pigeon completed multiple homing flights from three release sites, providing a large number of within-subject measurements with consistent speed-related effects across all individuals (Figure 2D and Figure 4A). The sample size was mainly constrained by the considerable difficulty of obtaining chronic wireless hippocampal recordings during natural flight. Nevertheless, the findings of this study should be regarded as proof-of-principle evidence, and studies with larger samples are needed to confirm the generalisability of these results.

## 5. Conclusions

In this study, we investigated the neural representation of flight speed in the pigeon’s HF during natural homing navigation. We observed that both the 50–70 Hz power spectral density ratio and the theta-demodulated amplitude in the HF systematically varied with flight speed, with these modulations remaining consistent across different release sites. Notably, multi-domain feature fusion significantly outperformed either single-feature in both speed state decoding and continuous speed regression, suggesting that hippocampal speed encoding relies on the integration of multiple spectral and temporal features rather than a single linear coder. In conclusion, our results provide evidence that hippocampal activity in pigeons carries stable, decodable information about flight speed during natural homing navigation. These LFP features are analogous to the speed-related signals described in the rodent Hp, but establishing whether similar information processing mechanisms exist will require further comparative work. Beyond fundamental neuroscience, these results may contribute to understanding neural cues relevant to racing pigeon performance. In addition, the demonstration of continuous speed encoding in a flying vertebrate may offer insights for bio-inspired navigation strategies in aerospace applications.

## Data Availability

The raw data supporting the conclusions of this article will be made available by the authors on request.

## Supplementary Materials

The following supporting information can be downloaded at: https://www.mdpi.com/article/10.3390/ani16162569/s1, Figure S1. Data acquisition device. The LFP acquisition module and IMU were mounted on the head, and the GPS module with battery were mounted on the back. Figure S2. Representative homing trajectories from six pigeons released from three spatially distinct sites.

## Abbreviations

CDF	cumulative distribution function
DAmp	theta-demodulated amplitude
HF	hippocampal formation
Hp	mammalian hippocampus
HS	high-speed flight state
LFPs	local field potentials
LS	low-speed flight state
MS	medium-speed flight state
NF	non-flight state
PSD	power spectral density
RMSE	root mean square error
