# Supplementary material for: Hippocampal Local Field Potentials Encode Continuous Flight Speed in Homing Pigeons via Complementary Gamma and Theta Signatures
Source: Animals (Basel). 2026 Aug 18;16(16):2569. doi: 10.3390/ani16162569 (PMC13508914; doi:10.3390/ani16162569)
Supplement: Supplementary file 1 [file animals-16-02569-s001.zip › animals-4466920-supplementary.pdf]

Surgery

Before electrode implantation, each bird was anaesthetised by intramuscular injection of 2% sodium pentobarbital (0.2 ml/100 g body weight). After a stable surgical plane of anaesthesia was achieved, the pigeon was placed in a prone position and secured in a custom-made stereotaxic apparatus (RWD Life Science, Shenzhen, China). Craniotomy was then performed for electrode implantation. Based on a stereotaxic atlas of the pigeon brain, the electrode implantation site targeting the HF was determined using the following coordinates: anteroposterior (AP), 4.50–6.50 mm; mediolateral (ML), 0.5–1.5 mm; and dorsoventral (DV), 1.00–1.50 mm. Four titanium screws were fixed around the implantation site and used as grounding connections. A recording electrode array was then implanted into the target region. The array consisted of a 2 × 4 arrangement of tungsten wires (Kedou Brain-Computer Technology, Suzhou, China). Given that the HF extends broadly along the sagittal axis, electrodes were implanted along this axis to ensure coverage of the target region and to obtain stable neural signals. Electrode locations were confirmed by histological verification (Fig. 1C). After implantation, the electrode array was secured with dental acrylic. During the first three days after surgery, the pigeons received cephalosporin antibiotics to reduce the risk of inflammation or infection and were closely monitored in individual cages with sufficient food and water. The pigeons were then returned to the loft and allowed to acclimate for at least 3 days before experiments began.

Data acquisition device

The device weighed 13.6 g and consisted of a neural signal acquisition module (1 kHz sampling), a global positioning system (GPS) module (10 Hz) and an inertial measure-ment unit (IMU, 100Hz). The neural signal acquisition module was based on an 8-channel ADS1299 chip (ADS1299IPAGR, Texas Instruments, Dallas, TX, USA) and recorded local field potentials (LFPs) at a sampling rate of 1 kHz. The GPS module (ATGM336H-5N) sampled position data at 10 Hz with a positioning accuracy of <2.5 m circular error prob-able. The IMU module (MPU6050) recorded inertial signals at a sampling rate of 100 Hz. The device was shown in Figure S1.

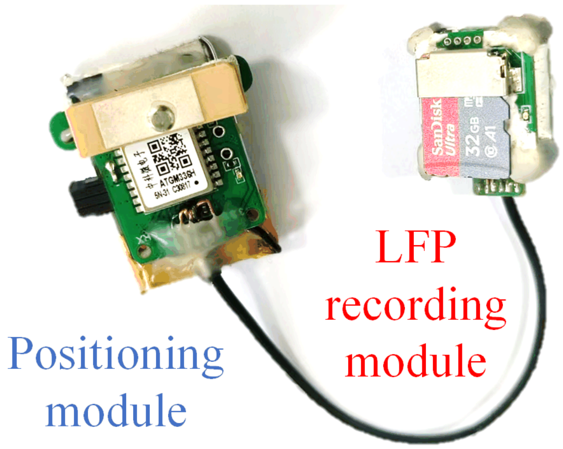

Figure S1. Data acquisition device. The LFP acquisition module and IMU were mounted on the head, and the GPS module with battery were mounted on the back.

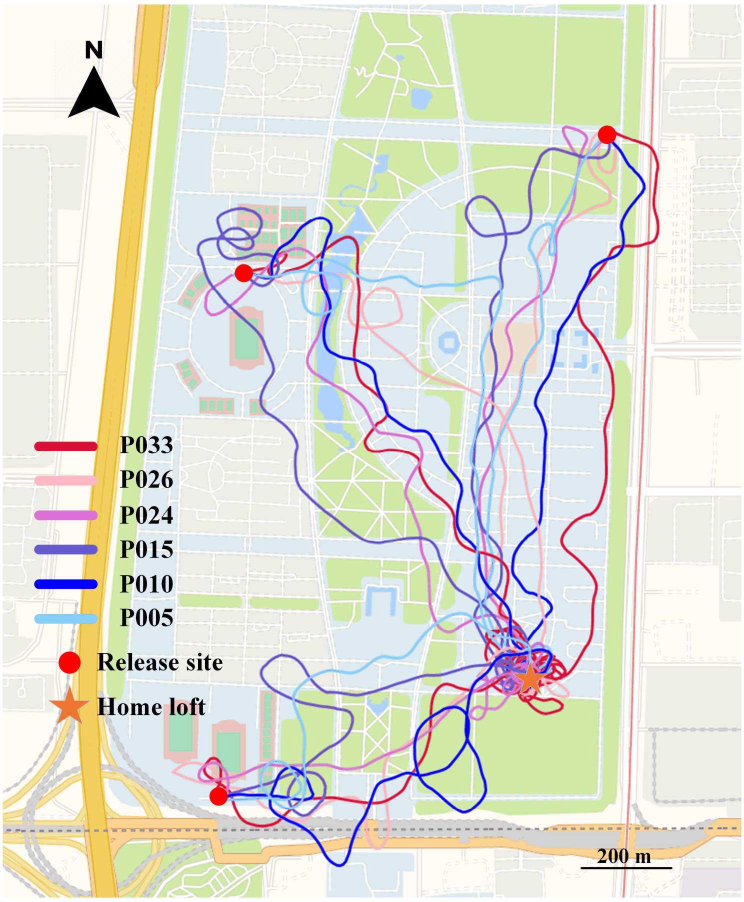

Figure S2. Representative homing trajectories from six pigeons released from three spatially distinct sites.
